# Risk Factors for MERS-CoV Seropositivity among Animal Market and Slaughterhouse Workers, Abu Dhabi, United Arab Emirates, 2014–2017

**DOI:** 10.3201/eid2505.181728

**Published:** 2019-05

**Authors:** Ahmed Khudhair, Marie E. Killerby, Mariam Al Mulla, Kheir Abou Elkheir, Wassim Ternanni, Zyad Bandar, Stefan Weber, Mary Khoury, George Donnelly, Salama Al Muhairi, Abdelmalik I. Khalafalla, Suvang Trivedi, Azaibi Tamin, Natalie J. Thornburg, John T. Watson, Susan I. Gerber, Farida Al Hosani, Aron J. Hall

**Affiliations:** Abu Dhabi Department of Health, Abu Dhabi, United Arab Emirates (A. Khudhair, M. Al Mulla, K.A. Elkheir, W. Ternanni, Z. Bandar, F. Al Hosani);; Sheikh Khalifa Medical City, Abu Dhabi (M.E. Killerby, S. Weber, M. Khoury, G. Donnelly);; Centers for Disease Control and Prevention, Atlanta, Georgia, USA (M.E. Killerby, S. Trivedi, A. Tamin, N.J. Thornburg, J.T. Watson, S.I. Gerber, A.J. Hall);; Abu Dhabi Food Control Authority, Abu Dhabi (S. Al Muhairi, A.I. Khalafalla)

**Keywords:** MERS-CoV, Middle East respiratory syndrome, MERS, coronavirus, camels, Abu Dhabi, United Arab Emirates, respiratory infections, seroepidemiology, risk factors, zoonoses, viruses

## Abstract

Camel contact is a recognized risk factor for Middle East respiratory syndrome coronavirus (MERS-CoV) infection. Because specific camel exposures associated with MERS-CoV seropositivity are not fully understood, we investigated worker–camel interactions and MERS-CoV seroprevalence. We assessed worker seroprevalence in 2 slaughterhouses and 1 live-animal market in Abu Dhabi, United Arab Emirates, during 2014–2017 and administered an epidemiologic survey in 2016 and 2017. Across 3 sampling rounds during 2014–2017, we sampled 100–235 workers, and 6%–19% were seropositive for MERS-CoV at each sampling round. One (1.4%) of 70 seronegative workers tested at multiple rounds seroconverted. On multivariable analyses, working as a camel salesman, handling live camels or their waste, and having diabetes were associated with seropositivity among all workers, whereas handling live camels and either administering medications or cleaning equipment was associated with seropositivity among market workers. Characterization of high-risk exposures is critical for implementation of preventive measures.

Middle East respiratory syndrome (MERS) coronavirus (MERS-CoV) was first identified as a cause of severe respiratory tract infections in Saudi Arabia in October 2012 (*1*). The clinical spectrum of MERS ranges from asymptomatic infection to acute respiratory distress syndrome and death (*2*). As of April 3, 2018, a total of 2,374 laboratory-confirmed cases of infection have been reported by 27 countries to the World Health Organization (WHO); the reported case-fatality rate is 35% (*2*). All reported cases have an epidemiologic link to the Arabian Peninsula, and imported cases have been reported in Europe, Asia, North America, and Africa. The United Arab Emirates has reported the third-highest number of MERS cases since 2012 (*3*).

MERS-CoV is a zoonotic virus, and dromedaries (camels) are recognized as a major virus reservoir for spillover to humans (*4*). Multiple studies have isolated MERS-CoV or MERS-CoV RNA from camels across the Arabian Peninsula and Africa (*5**–**11*). Serologic studies of camels in the Middle East and Africa have revealed MERS-CoV seroprevalence of >90%–97% (*8**,**11**–**13*). In natural infection, camels have been found to shed MERS-CoV in respiratory secretions and to a lesser extent in stool (*14**,**15*). Evidence of virus RNA has also been found in milk collected by traditional milking procedures, which involve calf suckling as a stimulus for milk letdown (*15*).

Epidemiologic links between infected camels and human MERS-CoV infections have been shown, with identical or nearly identical MERS-CoV genomes found in human cases and in camels with which they had direct contact (*16**–**18*). Also, a case–control study identified exposure to camels as a risk factor for human MERS-CoV infection (*19*). Human seroprevalence studies also support the association between MERS-CoV infection and camel contact; in Saudi Arabia MERS-CoV seroprevalence was found to be 15 times greater in camel shepherds and 23 times greater in slaughterhouse workers compared with the general population (*20*). Further studies have also shown high seroprevalence in specific occupational groups with various camel exposures (e.g., seropositivity was detected in 6.8% of a cohort of 294 camel workers in Qatar [*21*] and in 53% of a cohort of 30 camel workers in Saudi Arabia [*22*]).

Although multiple lines of evidence suggest camel exposure is associated with human MERS-CoV infection, the exact mechanisms of transmission are not fully understood. Information on specific risk factors relating to camel interactions are needed to further understand how the virus might be transmitted from camels to humans and to guide interventions to prevent zoonotic transmission, including changes to camel management practices. Because MERS-CoV vaccines are currently in development and have reported success in phase I clinical trials (*23*), knowledge of groups at risk for MERS-CoV infection might also be useful when considering future vaccine use. Our study aimed to identify risk factors for MERS-CoV seropositivity among live-animal market and slaughterhouse workers.

## Methods

### Study Site and Population

**Figure 1:**
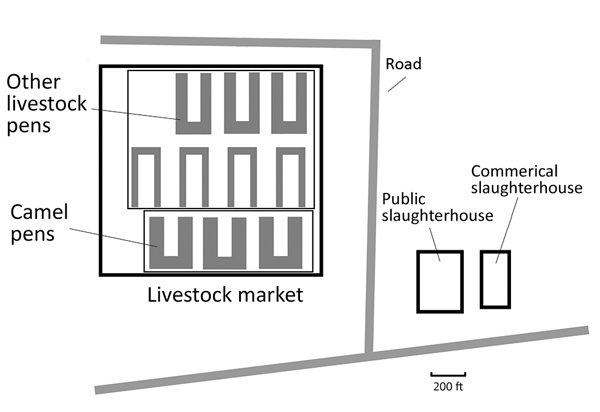
Diagram of study site indicating market and slaughterhouse settings, Abu Dhabi, United Arab Emirates.

The study sites consisted of an open-air animal market and 2 slaughterhouses (1 commercial and 1 public). All 3 facilities housed camels, goats, sheep, and cattle (Figure 1). Typically during the study period, approximately 460 persons worked at the market, 101 at the public slaughterhouse, and 29 at the commercial slaughterhouse. The market investigated in this study was linked to a human MERS case in 2015 (*24*). Prior investigation showed a large diversity of MERS-CoVs circulating among camels at the market; 109 (29%) of 276 screened camels had detectable MERS-CoV RNA in nasal swab specimens in the spring of 2015 (*25*).

### Serum Sampling and Data Collection

We conducted 3 rounds of worker serum sampling. The first round was conducted during May 11–14, 2014, and the second round during March 23–April 1 and May 7–13, 2015. During the first 2 rounds of sampling, all available workers at the market and public slaughterhouse were requested to provide a serum sample as part of a public health investigation. We conducted a third round of serum sampling during September 22–October 5, 2016, and March 20–23, 2017. The third round of sampling included workers at the market, public slaughterhouse, and the newly opened commercial slaughterhouse. All available workers were requested to provide serum samples, although participation was voluntary. Some, but not all, workers were repeatedly sampled, when feasible, during multiple rounds.

We administered an epidemiologic survey to all workers only during the third round of serum sampling in 2016 and 2017. No surveys were administered in 2014 or 2015. The survey consisted of questions covering worker demographics; occupational history; contact with various animal species; travel history; medical history; consumption of raw camel milk, raw camel meat, and camel urine; specific tasks performed with camels; types of personal protective equipment (PPE) worn; and handwashing practices (Appendix 1). Separate lists of questions covering specific camel tasks performed were asked of market and slaughterhouse workers because of the different nature of camel tasks among occupational groups. Interviews were conducted in Arabic by staff from the Abu Dhabi Department of Health.

### Laboratory Testing

Human serum samples were tested for MERS-CoV antibodies at the US Centers for Disease Control and Prevention (CDC) by using indirect ELISAs for nucleocapsid (N) and spike (S) proteins, followed by a confirmatory microneutralization test, as previously described (*26*). Samples were initially tested by using both N and S ELISAs as screening assays with serum diluted to 1:400. All serum samples with optical densities above assay cutoff were diluted serially, 4-fold, from 1:100 to 1:6,400, and used for endpoint titer determinations. Serum samples that were positive by N or S ELISA with titers at 1:400, 1:1600, or 1:6,400, plus 10% of samples negative by N or S ELISA at these titers, were tested by using microneutralization with live MERS-CoV performed in a Biosafety Level 3 laboratory, as previously described (*26*). In addition, we conducted confirmatory microneutralization tests on seronegative samples from any persons who showed a change in seropositivity status over time to confirm changes in seropositivity status. Samples were considered positive if positive on N and S ELISA or if positive on microneutralization. Specimens near the limits of detection but not consistently above or below these limits were considered indeterminate. For the epidemiologic analysis, persons with an indeterminate result were considered seronegative.

### Data Analyses

We used Epi Info 7 (https://www.cdc.gov/epiinfo) for data entry and R version 3.3.1 (https://cran.r-project.org/bin/windows/base/old/3.3.1) for data analysis. We performed comparisons between prevalence of work practices by setting (market vs. slaughterhouse) by using the Pearson χ^2^ square test. We used univariable logistic regression to estimate odds ratios, 95% CIs, and p values (Wald test) for all associations between potential risk factors and seropositivity. We assessed associations between demographics, occupational history, contact with various animal species, consumption of camel products, travel history, and medical history with seropositivity for all workers. We separately tested associations between specific interactions with camels, types of PPE worn, and handwashing practices with seropositivity for stratified subgroups of market and slaughterhouse workers because of the different nature of work setting and standard practices between these 2 populations. We then performed additional exploratory data description by occupation on the basis of results of univariable analyses.

We developed 3 multivariable logistic models to identify associations between risk factors and seropositivity. First, we constructed a model of risk factors common to all workers and then constructed occupationally stratified models (i.e., separate models for market workers and slaughterhouse workers) to model specific interactions with camels, PPE use, and handwashing practices. We combined or eliminated highly correlated variables, which were determined by condition indices and variance decomposition proportions. We reduced categorical variables to binary options if small group size was observed. We performed initial variable selection by using least absolute shrinkage and selection operator (LASSO) and then tested person-variable significance by using the likelihood ratio test with a cutoff of p<0.05 within an ordinary logistic regression model. We then included age and number of years worked at current setting as potential confounders in all 3 final models. We excluded persons with missing data at the LASSO stage but included them for the final logistic regression model.

For the stratified market worker and slaughterhouse models, we also included variables significant in the all workers model but not directly relating to camel interactions (e.g., reported underlying conditions) in the final occupationally stratified models. We did not include significant variables directly relating to camel exposures in the all workers model in the stratified models because more specific camel risk practices were assessed in the stratified models. For market and slaughterhouse models, we tested interactions between significant risk practices and select PPE use and handwashing practices for a protective effect.

## Results

### Serum Sample Results

**Figure 2:**
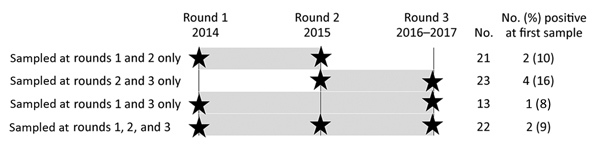
Number of workers sampled during >1 round of sampling (N = 79), Abu Dhabi, United Arab Emirates. Black stars indicate when serum samples were taken; gray shading indicates follow-up periods.

We sampled 100 workers in round 1 (2014), 151 workers in round 2 (2015), and 235 workers in round 3 (2016 and 2017); overall MERS-CoV seroprevalence was 6% for round 1, 19% for round 2, and 17% for round 3. Twenty-one persons had specimens taken at rounds 1 and 2, twenty-three at rounds 2 and 3, thirteen at rounds 1 and 3, and twenty-two at all 3 rounds (Figure 2). Of 70 persons who were seronegative at their first sample, only 1 (1.4%, 95% CI 0.1%–8.8%) seroconverted: a 30-year-old man who was a cleaner at the public slaughterhouse tested negative at round 1 and positive at round 2. Of 8 persons who were seropositive at their first sample, 1 (13%) was later found to be seronegative: a 28-year old man who was an administrative supervisor at the market was resampled between rounds 1 and 3. This person did not report handling camels or their waste and did not perform any tasks directly relating to camels. One additional person who had a positive serologic result at their first and second samples and an indeterminate result at their third sample was not subsequently evaluated for change in seropositive status. Because some study participants might have had different medical record numbers across the 3 sampling rounds, we could not determine all potential seroconversions or losses of seropositivity, although we also performed matching by name and age. We compiled serologic results for all participants who ever tested positive (Appendix 2).

### Epidemiologic Survey Results

In total, 235 persons both completed the epidemiologic survey and were sampled during round 3. One additional person completed the epidemiologic survey but refused serum sampling and was not included in any analyses. All 235 workers were men, and their median age was 35 years (range 19–64 years). The median number of years worked at the current settings was 6 (range 0.2–15 years). We observed no significant effect of age (p = 0.26) or years worked (p = 0.18) on seropositivity on univariable analysis. 

Worker occupations were categorized into animal handlers (n = 16), camel salesmen (n = 37), other animal salesmen (n = 41), animal or waste transporters (n = 27), butchers (n = 65), cleaners (n = 26), veterinarians (n = 9), and other (e.g., supervisor, cashier, and tourist guide) (n = 14). Salesmen only worked in the market, and butchers only worked in the slaughterhouses. The remaining occupations were found in both settings, but each person could only work at a slaughterhouse or the market. None of the workers reported working at any other job outside of the market or slaughterhouses, and the only animals reported present at home were poultry and stray cats.

Overall, 64 (44%) of 145 market workers had daily contact with camels or their waste, compared with 47 (52%) of 90 slaughterhouse workers (p = 0.28). Certain PPE use and handwashing were more frequently reported by slaughterhouse workers than market workers. Among slaughterhouse workers, 99% reported wearing a dust mask (equivalent to a surgical mask), compared with 21% of market workers (p<0.01). Only 37% of slaughterhouse workers reported taking their work clothes home, compared with 97% of market workers (p<0.01). Eighty-one percent of slaughterhouse workers reported washing their hands before and after each animal-related task, compared with 21% of market workers (p<0.01). Ninety-three percent of slaughterhouse workers reported washing their hands at the beginning and end of the day, compared with only 56% of market workers (p<0.01).

### Univariable Analyses

Rates of seropositivity were higher among market workers (29 [20%] of 145) than among slaughterhouse workers (11 [12%] of 90), although this difference was not statistically significant on univariable analysis (p = 0.17). By occupation, camel salesmen and animal or waste transporters had significantly higher odds of seropositivity than the reference group of other salesmen (Table 1).

**Table 1:** Characteristics of 235 market and slaughterhouse workers, by MERS-CoV serostatus, Abu Dhabi, United Arab Emirates*

Characteristic	Total no. participants	No. (%) participants				OR (95% CI)		p value
		Seronegative, n = 195		Seropositive, n = 40				
Work >50 h/wk	132	103 (78.0)		29 (22.0)		2.35 (1.14–5.17)		0.025
Worked another job in previous year	30	25 (83.3)		5 (16.7)		0.97 (0.31–2.53)		0.956
Occupation								
Other salesman	41	40 (97.6)		1 (2.4)		Ref		Ref
Animal handler	16	15 (93.8)		1 (6.3)		2.67 (0.1–70.37)		0.498
Butcher	65	56 (86.2)		9 (13.8)		6.43 (1.14–120.92)		0.083
Camel salesman	37	19 (51.4)		18 (48.6)		37.89 (7.03–707.16)		0.001
Cleaner	26	22 (84.6)		4 (15.4)		7.27 (1–147.06)		0.084
Animal or waste transporter	27	21 (77.8)		6 (22.2)		11.43 (1.79–223.48)		0.029
Veterinarian	9	8 (88.9)		1 (11.1)		5.00 (0.18–135.74)		0.272
Other	14	14 (100.0)		0		NA		0.990
Nationality								
Afghani	27	15 (55.6)		12 (44.4)		Ref		Ref
Bangladeshi	38	30 (78.9)		8 (21.1)		0.33 (0.11–0.97)		0.048
Pakistani	97	92 (94.8)		5 (5.2)		0.07 (0.02–0.21)		<0.001
Sudanese	38	25 (65.8)		13 (34.2)		0.65 (0.23–1.79)		0.404
Other	35	33 (94.3)		2 (5.7)		0.08 (0.01–0.32)		0.002
Contact with								
Camels or waste daily	111	78 (70.3)		33 (29.7)		7.07 (3.14–18.15)		<0.001
Cattle or waste daily	58	50 (86.2)		8 (13.8)		0.73 (0.29–1.61)		0.452
Goats or waste daily	88	79 (89.8)		9 (10.2)		0.43 (0.18–0.91)		0.036
Sheep or waste daily	89	80 (89.9)		9 (10.1)		0.42 (0.18–0.89)		0.031
Drank raw camel milk	25	18 (72.0)		7 (28.0)		2.09 (0.76–5.21)		0.129
Ate raw camel meat†	2	1 (50.0)		1 (50.0)		4.95 (0.19–126.94)		0.262
Travel								
To Saudi Arabia	4	4 (100.0)		0		NA		0.990
Within UAE	67	55 (82.1)		12 (17.9)		1.09 (0.5–2.25)		0.819
Underlying conditions								
Asthma	1	0		1 (100.0)		NA		0.985
Diabetes	9	5 (55.6)		4 (44.4)		4.22 (1–16.71)		0.038
Hypertension	12	9 (75.0)		3 (25.0)		1.68 (0.36–5.93)		0.455
Sought care for respiratory illness	45	36 (80.0)		9 (20.0)		1.28 (0.54–2.84)		0.555
Contact with anyone with respiratory illness	2	1 (50.0)		1 (50.0)		1.38 (0.09–7.84)		0.731
Had chest radiograph	13	10 (76.9)		3 (23.1)		1.5 (0.32–5.18)		0.552

*MERS-CoV, Middle East respiratory syndrome coronavirus; NA, not applicable; OR, odds ratio; Ref, referent; UAE, United Arab Emirates. †Total seronegative was 194 because answer from 1 person was missing. No workers ever contacted the following species: dogs, cats, bats, rodents, birds, and other animals; 1 worker reported rarely contacting horses.

Univariable analyses showed that several characteristics were associated with seropositivity among all workers (Table 1), including handling camels or their waste daily. Not all seropositive workers reported handling camels or their waste; 7 workers initially claimed they never handled camels or their waste, although 3 of these later reported that they contacted either camel equipment, viscera, or waste within the slaughterhouse. For the subgroup of market workers, univariable analyses revealed multiple camel exposures to be associated with seropositivity and 2 handwashing practices that were inversely associated with seropositivity (Table 2). For the subgroup of slaughterhouse workers, no individual risk factors were associated with seropositivity (Table 3).

**Table 2:** Comparison of practices among 145 MERS-CoV seronegative and seropositive market workers, Abu Dhabi, United Arab Emirates*

Characteristic	Total no. participants	No. (%) participants					OR (95% CI)			p value
		Seronegative, n = 116		Seropositive, n = 29						
Handle live camels	66	40 (60.6)		26 (39.4)			16.47 (5.38–72.06)			<0.001
Feed camels	51	31 (60.8)		20 (39.2)			6.09 (2.57–15.44)			<0.001
Clean camels	39	22 (56.4)		17 (43.6)			6.05 (2.56–14.83)			<0.001
Clean camel housing	37	19 (51.4)		18 (48.6)			8.35 (3.47–21.11)			<0.001
Handle camel waste	33	16 (48.5)		17 (51.5)			8.85 (3.63–22.56)			<0.001
Clean equipment	33	16 (48.5)		17 (51.5)			8.85 (3.63–22.56)			<0.001
Milk camels	18	12 (66.7)		6 (33.3)			2.26 (0.72–6.49)			0.138
Assist with camel birthing	27	18 (66.7)		9 (33.3)			2.45 (0.94–6.16)			0.060
Give medications to camels	35	18 (51.4)		17 (48.6)			7.71 (3.2–19.34)			<0.001
Contact with ill camel	37	20 (54.1)		17 (45.9)			6.8 (2.85–16.83)			<0.001
Wear dust mask and gloves	25	23 (92.0)		2 (8.0)			0.3 (0.05–1.1)			0.117
Wear respirator	0	0 (NA)		0 (NA)			NA			NA
Wear coveralls	15	13 (86.7)		2 (13.3)			0.59 (0.09–2.3)			0.500
Wear boots	3	3 (100.0)		0 (0.0)			NA			0.991
Who washes your clothes?										
Self	84	69 (82.1)		15 (17.9)			Ref			Ref
Household member	5	4 (80.0)		1 (20.0)			1.15 (0.06–8.49)			0.904
Other worker	56	43 (76.8)		13 (23.2)			1.39 (0.6–3.21)			0.439
Take work clothes home	141	113 (80.1)		28 (19.9)			0.74 (0.09–15.34)			0.801
Wash work clothes at home	89	73 (82.0)		16 (18.0)			0.72 (0.32–1.67)			0.444
Wash work clothes at workplace	6	3 (50.0)		3 (50.0)			4.35 (0.77–24.67)			0.082
Wash work clothes at laundry	63	50 (79.4)		13 (20.6)			1.07 (0.47–2.43)			0.867
Wash hands before and after each animal-related task	40	30 (75.0)		10 (25.0)			1.51 (0.61–3.56)			0.355
Wash hands at meal times	145	116 (80.0)		29 (20.0)			NA			NA
Wash hands at bathroom times	145	116 (80.0)		29 (20.0)			NA			NA
Wash hands at prayer times	145	116 (80.0)		29 (20.0)			NA			NA
Wash hands at beginning and end of day	81	70 (86.4)		11 (13.6)			0.4 (0.17–0.92)			0.033
Wash hands at toilets	143	114 (79.7)		29 (20.3)			NA			0.989
Wash hands at restaurant	64	50 (78.1)		14 (21.9)			1.23 (0.54–2.8)			0.616
Wash hands at mosque	69	55 (79.7)		14 (20.3)			1.04 (0.45–2.35)			0.934
Wash hands at barn	4	1 (25.0)		3 (75.0)			13.27 (1.63–274.19)			0.028

*MERS-CoV, Middle East respiratory syndrome coronavirus; OR, odds ratio.

**Table 3:** Comparison of practices among 90 MERS-CoV seronegative and seropositive slaughterhouse workers, Abu Dhabi, United Arab Emirates*

Characteristic	Total no. participants	No. (%) participants				OR (95% CI)				p value
		Seronegative, n = 79		Seropositive, n = 11						
Handle live camels	56	49 (87.5)		7 (12.5)		1.07 (0.3–4.37)				0.918
Perform antemortem exam of camels	6	5 (83.3)		1 (16.7)		1.48 (0.07–10.5)				0.732
Remove hide from camels†	52	46 (88.5)		6 (11.5)		0.83 (0.23–3.12)				0.780
Remove or handle viscera of camels‡	55	48 (87.3)		7 (12.7)		1.51 (0.39–7.4)				0.573
Clean equipment	68	58 (85.3)		10 (14.7)		3.62 (0.63–68.47)				0.233
Handle camel waste	57	50 (87.7)		7 (12.3)		1.01 (0.28–4.15)				0.982
Prepare cuts of camel meat	52	45 (86.5)		7 (13.5)		1.32 (0.37–5.39)				0.675
Conduct postmortem exam of camels	3	2 (66.7)		1 (33.3)		3.85 (0.17–43.92)				0.288
Slaughter camels	41	36 (87.8)		5 (12.2)		1 (0.27–3.57)				0.994
Contact with ill camel	3	2 (66.7)		1 (33.3)		3.85 (0.17–43.92)				0.288
Wear dust mask and gloves	88	77 (87.5)		11 (12.5)		NA				NA
Wear respirator	0	0		0		NA				NA
Wear coveralls	85	74 (87.1)		11 (12.9)		NA				NA
Wear boots	85	74 (87.1)		11 (12.9)		NA				NA
Who washes your clothes										
Self	30	28 (93.3)		2 (6.7)		Ref				Ref
Household member	2	0		2 (100.0)		NA				NA
Other worker	58	51 (87.9)		7 (12.1)		1.92 (0.43–13.49)				0.434
Take work clothes home	33	29 (87.9)		4 (12.1)		0.99 (0.24–3.55)				0.982
Wash work clothes at home	31	27 (87.1)		4 (12.9)		1.1 (0.27–3.98)				0.886
Wash work clothes at workplace	58	51 (87.9)		7 (12.1)		0.96 (0.27–3.93)				0.952
Wash work clothes at laundry	2	2 (100.0)		0		NA				NA
Wash hands before and after each animal-related task	73	64 (87.7)		9 (12.3)		1.05 (0.24–7.39)				0.949
Wash hands at meal times	89	78 (87.6)		11 (12.4)		NA				NA
Wash hands at bathroom times	90	79 (87.8)		11 (12.2)		NA				NA
Wash hands at prayer times	88	78 (88.6)		10 (11.4)		0.13 (0–3.41)				0.158
Wash hands at beginning and end of day	84	75 (89.3)		9 (10.7)		0.24 (0.04–1.9)				0.127
Wash hands at toilets	86	76 (88.4)		10 (11.6)		0.39 (0.05–8.4)				0.440
Wash hands at restaurant	0	0		0		NA				NA
Wash hands at mosque	3	3 (100.0)		0		NA				NA
Wash hands at basin	47	43 (91.5)		4 (8.5)		0.48 (0.12–1.71)				0.268

*Exam, examination; MERS-CoV, Middle East respiratory syndrome coronavirus, NA, not applicable; OR, odds ratio. †Total seronegative was 78 because answer from 1 person was missing. ‡Total seropositive was 10 because answer from 1 person was missing.

**Figure 3:**
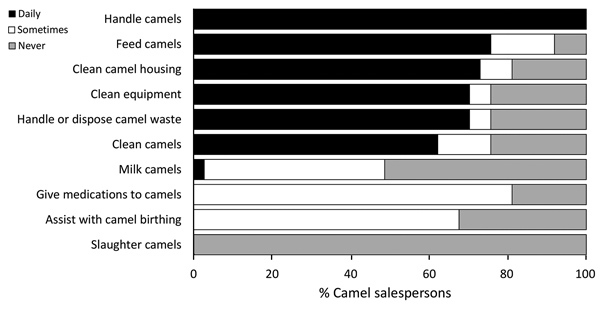
Frequency of tasks performed by camel salesmen (N = 37) in market, Abu Dhabi, United Arab Emirates.

Because camel salesmen had the highest odds of MERS-CoV seropositivity, we summarized their frequency of specific camel exposures separately (Figure 3). Direct observation of camel salesmen in the market showed that most of their time was spent in the camel pens, including while they ate and rested, and direct handling of the animals occurred frequently (data not shown).

### Multivariable Analyses

For the multivariable model evaluating risk factors associated with seropositivity in all workers, the following variables remained in the final logistic regression model: handling camels or their waste daily (adjusted odds ratio [aOR] 4.2, 95% CI 1.7–11.8), working as a camel salesman (aOR 4.0, 95% CI 1.6–10.1), and self-reported diabetes (aOR 6.2, 95% CI 1.2–30.3). All 3 factors significantly increased odds of seropositivity.

For market workers, multivariable analysis resulted in a final model in which the following variables were each independently associated with seropositivity: handling live camels (aOR 12.2, 95% CI 3.2–62.9), administering medications to camels (aOR 3.4, 95% CI 1.1–11.2), and self-reported diabetes (aOR 20.9, 95% CI 1.6–341.3). Cleaning equipment was also significantly associated with seropositivity (aOR 3.3, 95% CI 1.1–10.3); substituted for administering medication to camels, this factor produced a model with a near-identical fit along with the other risk factors. Given that administering medications to camels was highly correlated with cleaning equipment, the statistical significance of both factors was lost if both factors were included in the model because of collinearity (ρ = 0.65). None of the select PPE and handwashing practices evaluated as interactions with risk practices showed a significant protective effect. No individual risk factors were significantly associated with slaughterhouse workers by multivariable analysis.

## Discussion

Our study investigated risk factors for MERS-CoV seropositivity in animal market and slaughterhouse workers at a site previously associated with zoonotic transmission of MERS-CoV. Given the large number of camels present, including many young camels, and the mixing of camels from multiple sources, this site probably facilitates MERS-CoV transmission among camels. Our results demonstrated a relatively high MERS-CoV seroprevalence in workers at this site, ranging from 6% to 19% at each round across all occupations. Because we did not record occupation and other risk factors during the first 2 sampling rounds, we were unable to further assess reasons for the different seropositivity rates between sampling rounds.

We found particularly high seroprevalence in specific occupational groups, namely camel salesmen (49%) and animal or waste transporters (22%). Previous studies of workers with occupational exposure to camels have reported either lower seropositivity rates (e.g., 6.8% of 294 workers with occupational camel contact seropositive in Qatar [*21*] and 2.3% of 87 camel shepherds seropositive in Saudi Arabia [*20*]) or comparable seropositivity (e.g., 53% of camel workers positive in Saudi Arabia [*22*]). Our rates of seropositivity might underestimate actual exposure to MERS-CoV. Previous studies have demonstrated that examining MERS-CoV–specific T cells from MERS patients is more sensitive than examining serum antibodies alone (*27*). To examine T-cell responses, peripheral blood mononuclear cells must be collected, which was beyond the scope of our study.

On multivariable analysis, we found that contact with camels or their waste, working as a camel salesman, and self-reported diabetes were all independently associated with seropositivity in all workers. Because of small stratum size, belonging to other occupational groups could not be meaningfully explored as risk factors. Diabetes has previously been shown to be a commonly reported underlying condition in MERS cases (*28*), has been associated with risk for infection in a case–control study (*19*), and has been associated with increased risk for death in MERS patients (*29*). We found an association between diabetes and MERS-CoV seropositivity in a cohort with occupational exposure to camels. Although persons with diabetes might be at increased risk for MERS-CoV infection, the association between diabetes, MERS-CoV infection, and the resulting antibody response is still not fully understood. However, because persons with diabetes are considered at high risk for developing severe disease from MERS-CoV infection, WHO recommends these persons take precautions when visiting farms or markets where camels are present, including avoiding contact with camels (*3*).

Among market workers, handling live camels and either administering medications to camels or cleaning equipment were practices associated with significantly increased risk for MERS-CoV seropositivity. Given that administering medications to camels was highly correlated with cleaning equipment, neither factor was statistically significant if both were included in the model. The biological importance of these associations might therefore be difficult to interpret, because either or both risk factors could be statistically associated with MERS-CoV seropositivity and have an undefined strength of association. Practices potentially associated with camel calves, such as milking or assisting with camel birth, were not associated with MERS-CoV seropositivity despite a higher prevalence of viral RNA in camels <1 year of age compared with other ages (*30*) and a previously reported association between milking camels frequently and seropositivity (*31*). However, these practices were not commonly reported by market workers in our study, limiting the power to detect an association with seropositivity.

No specific work practices were found to be associated with seropositivity among slaughterhouse workers. Compared with market workers, slaughterhouse workers had less exposure to live camels and a higher self-reported prevalence of potentially protective practices such as PPE use and frequent handwashing. Although our multivariable analysis did not show a significant association between PPE use (e.g., wearing a dust mask and gloves) or handwashing practices and seropositivity, the small sample size might have restricted the power to detect interactions between PPE and camel exposures. Because camel-to-human transmission of MERS-CoV is not fully understood, WHO recommends broad preventive measures for slaughterhouse and market workers, including wearing facial protection when feasible, washing hands before and after each animal-related task, and washing soiled work clothes and shoes at the work place to avoid exposing family members to soiled work clothing (*3*). Where feasible, increased use of such measures could be encouraged, particularly in market workers, to decrease risk for infection.

Because only a single human MERS case has been reported in connection with the study site, our reported rates of seroprevalence suggest unrecognized transmission (and potentially unrecognized illness) at this site. However, because the length of time MERS-CoV antibodies persist is unknown (*32*), the time and place these infections might have occurred is unknown; transmission potential also exists in the United Arab Emirates outside of markets and slaughterhouses. Whether infections were symptomatic is also unknown. Participants were asked whether they had seen a healthcare provider for respiratory illness in the previous 12 months, but such reported illness was not associated with seropositivity, and multiple pathogens other than MERS-CoV could be responsible for any reported respiratory illness. Despite these limitations, MERS-CoV was detected in camels at the market during our study period (*25*), and an interim seroconversion was noted in 1 worker, suggesting active zoonotic transmission. Taken collectively, our findings suggest an underestimated prevalence of human MERS-CoV infection in settings where the virus is circulating among camels, probably resulting from camel-to-human transmission.

Our study had additional limitations, including the overall sample size and limited number of subjects within specific substrata. Concentration of camel interactions within particular occupational groups limited our ability to differentiate risk among specific camel interactions, despite our use of multivariable analysis. Furthermore, because most persons reported interactions either daily or never, determining whether increased risk was associated with increased frequency of individual tasks was not possible. Also, some MERS-CoV infections might not result in detectable antibodies, particularly when the infections are asymptomatic or mild (*32*). Persistence of detectable MERS-CoV antibodies after infection is not well-defined, limiting the ability of serologic testing to define previous infection. Finally, because of incomplete linkage of study participants by medical record numbers across the 3 sampling periods, not all potential seroconversions or losses of seropositivity could be determined.

In summary, our study found significantly increased odds of MERS-CoV seropositivity in persons with exposure to camels, in particular among those who handle live camels. Odds of seropositivity were also significantly higher for camel salesmen, suggesting that preventive measures such as PPE use could focus on specific occupational groups, in addition to individual work practices. Determining groups at highest risk for zoonotic MERS-CoV infection could also inform future vaccine trials in geographic regions where MERS-CoV is known to circulate.

Appendix 1 Questionnaire administered to animal market and slaughterhouse workers, Abu Dhabi, United Arab Emirates, 2014–2017.

Appendix 2 Serologic test results for all participants who ever tested seropositive for Middle East respiratory syndrome coronavirus, Abu Dhabi, United Arab Emirates, 2014–2017.
