# Correction: Vol. 25, No. 5

**DOI:** 10.3201/eid2506.C12506

**Published:** 2019-06

**Keywords:** errata, erratum, correction

The affiliation for Marie E. Killerby was listed incorrectly in Risk Factors for MERS-CoV Seropositivity among Animal Market and Slaughterhouse Workers, Abu Dhabi, United Arab Emirates, 2014–2017 (A. Khudhair et al.). Dr. Killerby is affiliated only with the Centers for Disease Control and Prevention (Atlanta, GA, USA). The article has been corrected online (https://wwwnc.cdc.gov/eid/article/25/5/18-1728_article).

