# Supplementary material for: Risk Factors for MERS-CoV Seropositivity among Animal Market and Slaughterhouse Workers, Abu Dhabi, United Arab Emirates, 2014–2017
Source: Emerg Infect Dis. 2019 May;25(5):927–35. doi: 10.3201/eid2505.181728 (PMC6478233; doi:10.3201/eid2505.181728)
Supplement: Appendix 1 — Questionnaire administered to animal market and slaughterhouse workers, Abu Dhabi, United Arab Emirates, 2014–2017. [file 18-1728-Techapp-s1.pdf]

Article DOI: https://doi.org/10.3201/eid2505.181728

Risk Factors for MERS-CoV Seropositivity among Animal Market and Slaughterhouse Workers, Abu Dhabi, United Arab Emirates, 2014–2017

Appendix 1

Market and Slaughterhouse Worker Surveys

Market Worker Survey

Form Serial Number: ER/BW/___ ___ ___/16

Interview Date: ________/________/________
Day Month Year

Interviewer initials: ___ ___ ___

Name of the animal market ______________________________

Location of the animal market (region and city) _________________________

<u>Subject Information</u>

FIRST NAME _________________________ LAST NAME _______________________

**AGE (years)**<br><br>**D.O.B: dd/mm/yyyy**	*GENDER* ☐ *Male* ☐ *Female*<br><br>*NATIONALITY* ______________________________

CONSENTED

☐ **YES** ☐ **NO**

BLOOD SPECIMEN COLLECTED

☐ **YES** ☐ **NO**

Specimen collection date:

________/________/________
Day Month Year

Investigator Notes:

Sample Label:
(Place sticker here)

Home phone ______________________ Mobile phone 1______________________

Mobile phone 2 ________________________

CURRENT HOME ADDRESS

Street ____

City_________________ Area: _____________________

INTERNAL USE:
Data Entered: **Data Entry Checked:**
Initials: **Initials:**

OCCUPATION

What is your job title at the animal market? ______________________________________

Do you have animals at your home? ☐ **YES** ☐ **NO**

If YES, specify type and number of animals at home: ___________________________

How long have you worked at this animal market? ________years _______months

If less than one year, have you worked at any other animal markets prior to this one?

☐ **YES** (specify location, number, or name of the animal market) _________________

☐ **NO**

How many hours per week do you typically work at the animal market? ______hours/week

How many hours per week do you typically work with animals outside the animal market? ____ hours/week

Do you currently hold other jobs aside from working at the animal market? ☐**YES** ☐**NO**

If YES, Please specify location and job title of other job(s): _________________________

____

How often do you perform each of the following?

Handle camels	☐ **NEVER**	☐ **SOMETIMES**	☐ **DAILY**
Feed camels	☐ **NEVER**	☐ **SOMETIMES**	☐ **DAILY**
Clean camels	☐ **NEVER**	☐ **SOMETIMES**	☐ **DAILY**
Clean camel housing	☐ **NEVER**	☐ **SOMETIMES**	☐ **DAILY**
Handle or dispose camel waste	☐ **NEVER**	☐ **SOMETIMES**	☐ **DAILY**
Clean equipment	☐ **NEVER**	☐ **SOMETIMES**	☐ **DAILY**
Milk camels	☐ **NEVER**	☐ **SOMETIMES**	☐ **DAILY**
Slaughter camels	☐ **NEVER**	☐ **SOMETIMES**	☐ **DAILY**
Assist with camel parturition (birthing)	☐ **NEVER**	☐ **SOMETIMES**	☐ **DAILY**
Give medications/vaccines to camels	☐ **NEVER**	☐ **SOMETIMES**	☐ **DAILY**

Other tasks (specify and indicate frequency):

ANIMAL EXPOSURES

In the past 12 months, have you had direct physical contact with any animals or their waste?

Camels	☐ **Never**	☐ **Rarely**	☐ **Monthly**	☐ **Weekly**	☐ **DAILY**
Goats	☐ **Never**	☐ **Rarely**	☐ **Monthly**	☐ **Weekly**	☐ **DAILY**
Sheep	☐ **Never**	☐ **Rarely**	☐ **Monthly**	☐ **Weekly**	☐ **DAILY**
Cattle	☐ **Never**	☐ **Rarely**	☐ **Monthly**	☐ **Weekly**	☐ **DAILY**
Horses	☐ **Never**	☐ **Rarely**	☐ **Monthly**	☐ **Weekly**	☐ **DAILY**
Dogs	☐ **Never**	☐ **Rarely**	☐ **Monthly**	☐ **Weekly**	☐ **DAILY**
Cats	☐ **Never**	☐ **Rarely**	☐ **Monthly**	☐ **Weekly**	☐ **DAILY**
Bats	☐ **Never**	☐ **Rarely**	☐ **Monthly**	☐ **Weekly**	☐ **DAILY**
Rodents	☐ **Never**	☐ **Rarely**	☐ **Monthly**	☐ **Weekly**	☐ **DAILY**
Birds	☐ **Never**	☐ **Rarely**	☐ **Monthly**	☐ **Weekly**	☐ **DAILY**

Other animals (specify and indicate frequency):

Were any of the animals you had contact with ill? ☐ **YES** ☐ **NO** ☐ **Do not recall**

If YES, please describe the following:

Which animals were ill? ____

What were their symptoms? ____

Did you develop any illness after this contact with the ill animal? ☐ **YES** ☐ **NO**

If YES, what were your symptoms? ______________________________________

AND how long after this contact did your symptoms begin?
☐ **<1 Week** ☐ **1-2 Weeks** ☐ **3-4 Weeks** ☐ **>1 Month**

In the past 12 months, have you eaten any raw camel milk or milk products?
☐ **YES** ☐ **NO** ☐ **Do not recall**

In the past 12 months, have you drunk camel urine?
☐ **YES** ☐ **NO** ☐ **Do not recall**

In the past 12 months, have you eaten uncooked camel meat?
☐ **YES** ☐ **NO** ☐ **Do not recall**

PPE and HYGIENE

Do you usually wear personal protective equipment (PPE) when handling animals or their waste?

☐ **YES** ☐ **NO**

If YES, check all that apply:

☐ **Gloves** ☐ **Dust Mask** ☐ **Respirator** ☐ **Coveralls** ☐ **Boots or boot covers**

☐ **Eye protection (googles, safety glasses)** ☐ **Other (specify):**

Do you take your work clothes home with you? ☐ **YES** ☐ **NO**

Where are your work clothes washed? ☐ **Home** ☐ **Work place** ☐ **Other (specify)**

Who washes your work clothes? ☐ **Self** ☐ **Household member** ☐ **Other worker**

At which of the following times do you usually wash your hands at the animal market (check all that apply)?

☐ Before and after each animal-related task

☐ At meal times

☐ At bathroom times

☐ At prayer times

☐ At the beginning and end of the day

Where do you wash your hands at the animal market? ___________________________________

TRAVEL HISTORY

During the last 12 months, did you visit: ☐ **YES** ☐ **NO** ☐ do not recall

If yes, check all that apply:

☐ Bahrain	☐ Palestinian Territories	☐ Lebanon
☐ Iraq	☐ Qatar	☐ Yemen
☐ Iran	☐ Saudi Arabia	☐ Oman
☐ Israel	☐ Syria	☐ Jordan
☐ Kuwait	☐ South Korea	☐ Other (list)_______________

Did you travel within United Arab Emirates? ☐ **YES** ☐ **NO**

If YES, where?____

ILLNESS HISTORY

During the last 12 months, have you seen a doctor for a respiratory illness? (for symptoms including any of the following: cough, fever, runny nose, shortness of breath, rapid or shallow breathing, sore throat, vomiting after cough, and wheezing):

☐ **YES** ☐ **NO** ☐ **Do not recall**

If YES…

Where did you seek care for this illness (name/location of medical institution)?

____

Can you describe your symptoms during this time?

____

What medications did you take during this time?

____

Was a chest x-ray performed?

☐ **YES** ☐ **NO** ☐ **Do not recall**

If YES,

Health insurance card number ___________

Medical record number _______________

Do you have any underlying medical conditions (e.g. immunocompromised, heart disease, pulmonary disease (COPD, emphysema, cancer, diabetes, hypertension, smoking, etc.)?

☐ **YES** ☐ **NO**

If YES, please describe ____

HUMAN EXPOSURES

Did you have contact with anyone hospitalized for respiratory illness during the last 12 months?

☐ **YES** ☐ **NO** ☐ **Do not recall**

ADDITIONAL NOTES:

Slaughterhouse Worker Survey

Form Serial Number: ER/BW/___ ___ ___/16

Interview Date: ________/________/________
Day Month Year

Interviewer initials: ___ ___ ___

Name of the slaughter house ______________________________

Location of the slaughter house (region and city) _________________________

Subject Information

FIRST NAME _________________________ LAST NAME _______________________

AGE (years)

D.O.B: dd/mm/yyyy

GENDER ☐ *Male* ☐ *Female*

NATIONALITY ______________________________

CONSENTED

☐ **YES** ☐ **NO**

BLOOD SPECIMEN COLLECTED

☐ **YES** ☐ **NO**

Specimen collection date:

________/________/________
Day Month Year

Investigator Notes:

Sample Label:
(Place sticker here)

Home phone _______________________ Mobile phone 1_______________________

Mobile phone 2 _________________________

CURRENT HOME ADDRESS

Street ____

City__________________ Area: ______________________

INTERNAL USE:

Data Entered:
Initials:

Data Entry Checked:
Initials:

ANIMAL EXPOSURES

In the past 12 months, have you had direct physical contact with any animals or their waste?

Camels	☐ **Never**	☐ **Rarely**	☐ **Monthly**	☐ **Weekly**	☐ **DAILY**
Goats	☐ **Never**	☐ **Rarely**	☐ **Monthly**	☐ **Weekly**	☐ **DAILY**
Sheep	☐ **Never**	☐ **Rarely**	☐ **Monthly**	☐ **Weekly**	☐ **DAILY**
Cattle	☐ **Never**	☐ **Rarely**	☐ **Monthly**	☐ **Weekly**	☐ **DAILY**
Horses	☐ **Never**	☐ **Rarely**	☐ **Monthly**	☐ **Weekly**	☐ **DAILY**
Dogs	☐ **Never**	☐ **Rarely**	☐ **Monthly**	☐ **Weekly**	☐ **DAILY**
Cats	☐ **Never**	☐ **Rarely**	☐ **Monthly**	☐ **Weekly**	☐ **DAILY**
Bats	☐ **Never**	☐ **Rarely**	☐ **Monthly**	☐ **Weekly**	☐ **DAILY**
Rodents	☐ **Never**	☐ **Rarely**	☐ **Monthly**	☐ **Weekly**	☐ **DAILY**
Birds	☐ **Never**	☐ **Rarely**	☐ **Monthly**	☐ **Weekly**	☐ **DAILY**

Other animals (specify and indicate frequency):

Were any of the animals you had contact with ill? ☐ **YES** ☐ **NO** ☐ **Do not recall**

If YES, please describe the following:

Which animals were ill? ____

What were their symptoms? ____

Did you develop any illness after this contact with the ill animal? ☐ **YES** ☐ **NO**

If YES, what were your symptoms? __________________________________

AND how long after this contact did your symptoms begin?

☐ **<1 Week** ☐ **1-2 Weeks** ☐ **3-4 Weeks** ☐ **>1 Month**

In the past 12 months, have you eaten any raw camel milk or milk products?

☐ **YES** ☐ **NO** ☐ **Do not recall**

In the past 12 months, have you drunk camel urine?

☐ **YES** ☐ **NO** ☐ **Do not recall**

In the past 12 months, have you eaten uncooked camel meat?

☐ **YES** ☐ **NO** ☐ **Do not recall**

PPE and HYGIENE

Do you usually wear personal protective equipment (PPE) when handling animals or their waste?

☐ **YES** ☐ **NO**

If YES, check all that apply:

☐ **Gloves** ☐ **Dust Mask** ☐ **Respirator** ☐ **Coveralls** ☐ **Boots or boot covers**

☐ **Eye protection (googles, safety glasses)** ☐ **Other (specify):**

Do you take your work clothes home with you? ☐ **YES** ☐ **NO**

Where are your work clothes washed? ☐ **Home** ☐ **Work place** ☐ **Other (specify)**

Who washes your work clothes? ☐ **Self** ☐ **Household member** ☐ **Other worker**

At which of the following times do you usually wash your hands at the slaughter house (check all that apply)?

☐ Before and after each animal-related task

☐ At meal times

☐ At bathroom times

☐ At prayer times

☐ At the beginning and end of the day

Where do you wash your hands at the slaughter house? ___________________________________

TRAVEL HISTORY

During the last 12 months, did you visit: ☐ **YES** ☐ **NO** ☐ do not recall

If yes, check all that apply:

☐ Bahrain	☐ Palestinian Territories	☐ Lebanon
☐ Iraq	☐ Qatar	☐ Yemen
☐ Iran	☐ Saudi Arabia	☐ Oman
☐ Israel	☐ Syria	☐ Jordan
☐ Kuwait	☐ South Korea	☐ Other

(list)______________

Did you travel within United Arab Emirates? ☐ **YES** ☐ **NO**

If YES, where?____

ILLNESS HISTORY

During the last 12 months, have you seen a doctor for a respiratory illness? (for symptoms including any of the following: cough, fever, runny nose, shortness of breath, rapid or shallow breathing, sore throat, vomiting after cough, and wheezing):

☐ **YES** ☐ **NO** ☐ **Do not recall**

If YES…

Where did you seek care for this illness (name/location of medical institution)?

____

Can you describe your symptoms during this time?

____

What medications did you take during this time?

____

Was a chest x-ray performed?

☐ **YES** ☐ **NO** ☐ **Do not recall**

If YES,

Health insurance card number ___________

Medical record number ________________

Do you have any underlying medical conditions (e.g. immunocompromised, heart disease, pulmonary disease (COPD, emphysema, cancer, diabetes, hypertension, smoking, etc.)?

☐ **YES** ☐ **NO**

If YES, please describe ____

HUMAN EXPOSURES

Did you have contact with anyone hospitalized for respiratory illness during the last 12 months?

☐ **YES** ☐ **NO** ☐ **Do not recall**

ADDITIONAL NOTES:
