# Supplementary material for: Risk Factors for MERS-CoV Seropositivity among Animal Market and Slaughterhouse Workers, Abu Dhabi, United Arab Emirates, 2014–2017
Source: Emerg Infect Dis. 2019 May;25(5):927–35. doi: 10.3201/eid2505.181728 (PMC6478233; doi:10.3201/eid2505.181728)
Supplement: Appendix 2 — Serologic test results for all participants who ever tested seropositive for Middle East respiratory syndrome coronavirus, Abu Dhabi, United Arab Emirates, 2014–2017. [file 18-1728-Techapp-s2.pdf]

Article DOI: https://doi.org/10.3201/eid2505.181728

Risk Factors for MERS-CoV Seropositivity among Animal Market and Slaughterhouse Workers, Abu Dhabi, United Arab Emirates, 2014–2017

Appendix 2

Appendix 2 Table. Results of N ELISA, S ELISA, and microneutralization tests for all participants who ever had a seropositive test result*

Participant ID	Sample number	Year of collection	Sampling round	OD N ELISA at 1:400 cutoff 0.29	OD S ELISA at 1:400 cutoff 0.2	MNT (cutoff 20)	Interpretation†
1	1st	2014	1	1.57	0.32	40	Positive
2	1st	2014	1	1.54	0.26	20	Positive
3	1st	2014	1	0.64	0.33	80	Positive
4	1st	2014	1	0.79	0.04	20	Positive
5	1st	2014	1	1.12	0.19	20	Positive
6	1st	2014	1	1.11	0.31	80	Positive
7	1st	2015	2	0.21	0.18	40	Positive
8	1st	2014	1	0.22	0.01	<20	Negative
5	2nd	2015	2	0.35	0.14	20	Positive
6	2nd	2015	2	0.40	0.24	40	Positive
3	2nd	2015	2	0.22	0.34	40	Positive
8	1st	2015	2	1.16	0.37	40	Positive
8	1st	2015	2	0.45	0.20	20	Positive
9	1st	2015	2	0.78	1.37	80	Positive
10	1st	2015	2	0.15	0.48	80	Positive
11	1st	2015	2	0.15	0.33	40	Positive
12	1st	2015	2	0.83	0.28	20	Positive
13	1st	2015	2	0.86	0.20	40	Positive
14	1st	2015	2	0.03	0.68	20	Positive
15	1st	2015	2	0.48	0.26	40	Positive
16	1st	2015	2	0.76	0.37	40	Positive
17	1st	2015	2	0.38	0.86	20	Positive
18	1st	2015	2	0.40	0.28	160	Positive
19	1st	2015	2	0.48	0.94	80	Positive
20	1st	2015	2	0.56	0.56	80	Positive
21	1st	2015	2	0.58	0.54	40	Positive
22	1st	2015	2	0.34	0.54	40	Positive
23	1st	2015	2	0.13	0.32	40	Positive
24	1st	2015	2	0.44	0.16	20	Positive
25	1st	2015	2	0.69	0.35	20	Positive
26	1st	2015	2	0.72	0.45	40	Positive
27	1st	2015	2	0.03	0.92	320	Positive
28	1st	2015	2	0.65	0.22	20	Positive
29	1st	2015	2	0.49	0.67	40	Positive
30	1st	2015	2	0.64	0.28	80	Positive
2	2nd	2015	2	1.33	0.62	<20	Positive
5	3rd	2016	3	0.26	0.09	<20	Indeterminate‡
7	2nd	2016	3	0.09	0.10	20	Positive
31	1st	2016	3	1.01	0.36	160	Positive
32	1st	2016	3	0.13	0.80	20	Positive
33	1st	2016	3	0.29	0.87	40	Positive
34	1st	2016	3	0.07	0.64	40	Positive
35	1st	2016	3	0.43	1.14	40	Positive
36	1st	2016	3	0.04	0.34	20	Positive
37	1st	2016	3	0.02	0.88	80	Positive
38	1st	2016	3	0.19	0.85	40	Positive
39	1st	2016	3	0.39	0.93	20	Positive
40	1st	2016	3	0.05	1.39	20	Positive
2	3rd	2016	3	0.36	0.94	20	Positive

Participant ID	Sample number	Year of collection	Sampling round	OD N ELISA at 1:400 cutoff 0.29	OD S ELISA at 1:400 cutoff 0.2	MNT (cutoff 20)	Interpretation†
25	2nd	2016	3	0.00	0.04	80	Positive
21	2nd	2016	3	0.25	0.59	20	Positive
41	1st	2016	3	0.52	0.48	<20	Positive
4	2nd	2016	3	0.01	0.02	<20	Negative
42	1st	2016	3	0.80	0.76	<20	Positive
43	1st	2017	3	1.33	0.74	<20	Positive
44	1st	2017	3	0.55	0.33	<20	Positive
45	1st	2017	3	0.02	0.34	20	Positive
46	1st	2017	3	0.46	0.98	80	Positive
16	2nd	2017	3	0.65	0.79	160	Positive
47	1st	2017	3	0.53	0.60	80	Positive
48	1st	2017	3	1.60	0.70	640	Positive
49	1st	2017	3	0.24	0.53	20	Positive
50	1st	2017	3	0.39	0.46	20	Positive
51	1st	2017	3	0.13	0.27	20	Positive
52	1st	2017	3	0.42	0.46	20	Positive
53	1st	2017	3	0.38	0.46	40	Positive
54	1st	2017	3	0.08	0.43	40	Positive
55	1st	2017	3	0.22	0.28	20	Positive
56	1st	2017	3	0.49	0.72	40	Positive
57	1st	2017	3	0.40	0.75	20	Positive
58	1st	2017	3	0.58	0.87	160	Positive
59	1st	2017	3	0.53	0.33	40	Positive
60	1st	2017	3	0.24	0.27	40	Positive
61	1st	2017	3	0.66	1.02	80	Positive
62	1st	2017	3	0.47	0.79	160	Positive
63	1st	2017	3	0.20	0.51	20	Positive
64	1st	2017	3	0.08	0.60	20	Positive
65	1st	2017	3	0.12	0.34	20	Positive

*Cell shading indicates ELISA results above the cutoff point.

†Samples were considered positive if positive on N and S ELISA, or if positive on microneutralization. Specimens near the limits of detection but not consistently above or below these limits were considered indeterminate. For the epidemiologic analysis, individuals with an indeterminate result were considered seronegative.

‡Participant 5 was considered negative for epidemiologic analysis during sampling round 3, but as this indeterminate second sample result was reported after a positive result one year prior, participant 5 was not considered to have lost seropositive status.
